# Dexmedetomidine vs Propofol as an Adjunct to Ketamine for Electroconvulsive Therapy Anaesthesia

**DOI:** 10.5152/TJAR.2021.21217

**Published:** 2022-04-01

**Authors:** Tuğçe Yeter, Aybike Onur Gönen, Ercan Türeci

**Affiliations:** 1 Department of Anaesthesiology and Reanimation, Dr. Sadi Konuk Training and Research Hospital, İstanbul, Turkey; 2 Department of Anaesthesiology and Reanimation, İstanbul University Cerrahpaşa Faculty of Medicine, İstanbul, Turkey

**Keywords:** Dexmedetomidine, electroconvulsive therapy, ketamine, none-or anaesthesia, outpatient anaesthesia, propofol

## Abstract

**Objective:**

Electroconvulsive therapy is an effective non-pharmacological treatment for refractory mental illness, where a generalized seizure is induced under general anaesthesia. An ideal combination of the anaesthetic drugs should keep the patient paralyzed and unconscious for a few minutes, while allowing rapid recovery, supporting peri-procedural hemodynamic and respiratory stability, and permitting an effective treatment. We examined whether dexmedetomidine is advantageous over propofol as an adjunct to ketamine during electroconvulsive therapy.

**Methods:**

Sixty patients were randomly assigned to receive either ketamine-propofol or ketamine-dexmedetomidine. Periprocedural hemodynamic and respiratory parameters, recovery metrics, seizure length, side effects, and cost of treatment were compared between the 2 groups.

**Results:**

Hemodynamic response, respiratory status, and side effect profiles in ketamine-dexmedetomidine and ketamine-propofol groups were similar. Ketamine-dexmedetomidine combination showed a slight advantage with returning to baseline mean arterial pressure levels sooner. Seizures lasted longer in ketamine-dexmedetomidine group (41.8 seconds vs 25.4 seconds, *P * = .001). Recovery time was similar in 2 groups (*P*  = .292); however, time to eye opening and following orders was longer in ketamine-dexmedetomidine (*P* < .001 and *P*  = .003). The cost of treatment for ketamine-dexmedetomidine was much higher than ketamine-propofol (*P* < .001).

**Conclusions:**

Ketamine-dexmedetomidine induction led to longer seizures during electroconvulsive therapy compared to ketamine-propofol. We observed slightly better hemodynamic stability with dexmedetomidine compared to propofol. Despite dexmedetomidine’s disadvantages with a longer duration of administration, possible higher cost, and minor delay in initial recovery, it should be considered as a feasible agent for electroconvulsive therapy anaesthesia.

**Main Points**

- General anaesthesia is an essential component of electroconvulsive therapy treatment. As ketamine is gaining popularity in psychiatric treatments, adjuncts to use ketamine should be considered.
- Combining ketamine with dexmedetomidine led to longer seizures than with propofol.
- Hemodynamic stability was better with ketamine-dexmedetomidine compared to ketamine-propofol.
- Recovery is slightly longer with dexmedetomidine than with propofol; however, discharge to ward time remains the same.

## Introduction

Electroconvulsive therapy (ECT) is an effective treatment option for treatment-resistant severe psychiatric illnesses. An electric shock-induced generalized seizure alters brain biochemistry and physiology in a way that alleviates severe depressive and psychotic symptoms.^1,2^ Electroconvulsive therapy is always performed under general anaesthesia to provide the best and safest treatment experience for the patient.^2^

Electroconvulsive therapy is one of the most effective treatments in psychiatry.^2^ Individual success of treatment depends on several factors that have not yet been clearly identified. Although the length of the seizure has not been linked to treatment success, seizure duration of at least 15-25 seconds is desirable.^3^ If a shorter seizure is observed, a second seizure is induced with measures to prolong its duration or voltage is increased.^3^ In addition to patient and procedural factors, the anaesthetic drugs also affect the seizure activity. Hence, anaesthetic management may be contributory to the treatment outcome.^4^

Ideal anaesthetic drug combination for an ECT case should be fast- and short-acting.^5,6^ Complete unconsciousness and neuromuscular blockade should be achieved for patients’ comfort and well-being. If not intervened, physical activity caused by the generalized seizure can lead to soft tissue damage, bone fractures, and even nerve palsies. Patients should remain hemodynamically stable and recover quickly without any anaesthesia-related side effects, such as respiratory depression. Anaesthetic drugs should not suppress seizure activity.^4^ It is challenging to find a drug or drug combination that will hit all these points in all patients, therefore extensive comparison of numerous drugs and their combinations are available in medical literature.^5,6^

Ketamine is a non-competitive N-methyl-D-aspartate (NMDA) agonist and is unique among common anaesthetic drugs with its dissociative profile.^7^ It provides analgesia and amnesia while patients’ muscle tone, respiratory drive, and cardiovascular functions remain unsuppressed.^7^ Ketamine increases the sympathetic tone on the cardiovascular system, which may be disadvantageous in the setting of ECT.^8^ Intravenous ketamine use provides quick anaesthesia and may also lengthen seizure duration in ECT.^6^

Propofol is a short-acting intravenous hypnotic and potentiates the inhibitory activity of Gamma-aminobutyric acid -A (GABA-A) receptors.^7^ It achieves loss of consciousness and apnea quickly and is commonly used during ECT despite its anticonvulsant activity.^9^ Studies show that ECT can remain effective with its use and that propofol suppresses the hemodynamic response to the seizure.^6^ Its short duration of action with a single bolus dose allows for a smooth post-ECT recovery.^9^ Lowest dose possible should ideally be used for longer seizure activity.

Dexmedetomidine is an alpha-2 adrenergic receptor agonist.^7^ Its intravenous administration leads to anxiolysis, sedation, hypnosis, and analgesia without respiratory suppression.^7^ It can also decrease the sympathetic tone over the cardiovascular system. Its inaction over the seizure activity is ideal for ECT.^4^ We expect that dexmedetomidine would attenuate ketamine’s hemodynamic effects without undermining respiratory stability or quality of seizure activity.

The combination of ketamine and propofol has been studied for ECT extensively. Although better physiological and treatment outcomes in terms of seizure duration are observed, better clinical outcome is not proven yet.^8,10-12^ In this study, we compared ketamine-dexmedetomidine combination (KD) to ketamine-propofol (KP) in the search for the probability of a better anaesthetic recipe for ECT.

## Methods

Sixty patients, aged 18-60 years, were enrolled in this prospective, randomized, controlled study after approval of İstanbul University Cerrahpaşa Faculty of Medicine Clinical Research Ethics Committee (November 14, 2018—72109855-604.01.01-92505). Informed written consent was obtained from the patients or their legal guardians if the patient did not have the capacity to consent. Exclusion criteria included being American Society of Anesthesiologists class III or more, pregnancy, organ failure, alcohol or other drug addictions, lithium use, low pseudocholinesterase levels, receiving first session of ECT, and having glaucoma.

All patients had their anaesthetic evaluation at the preoperative anaesthesia clinic. After patients arrived to the ECT room, monitors for non-invasive blood pressure, electrocardiogram, and peripheral oxygen saturations were applied. A 20-gauge venous cannula was placed on the right arm, freeing the left arm for observing seizure activity with tourniquet application.

Initial measurements of systolic arterial pressure (SAP), diastolic arterial pressure (DAP), mean arterial pressure (MAP), heart rate (HR), and peripheral oxygen saturation (SpO_2_) were recorded before any drug administration (baseline). Patients were randomly assigned to KD (n = 30) and (KP (n = 30) groups with the closed envelope technique. Measurements for a single ECT session were recorded for each patient and each patient was enrolled in the study only once. 

Ketamine-propofol patients received 1 mg kg^−1^ intravenous (iv) propofol (Propofol Fresenius Kabi Avusturya GmbH, Avusturya) injected over 1 minute and a bolus dose of 1 mg kg^−1^ iv ketamine (Ketalar, Pfizer, Lüleburgaz, Turkey), KD patients received 1 µg kg^-1^ iv dexmedetomidine (Precedex, Abbott, North. Chicago, IL, USA) injected over 10 minutes and a bolus dose of 1 mg kg^-1^ iv ketamine. After the anesthesiologist administered these drugs, the psychiatric team was called into the room for ECT. If the patient needed extra anaesthetic, we planned to administer 0.5 mg kg^−1^ ketamine. After loss of consciousness, the tourniquet on the left upper arm was inflated to a pressure of 50 mm Hg above the SAP. Neuromuscular blockade (NMB) was achieved with iv injection of 1 mg kg^−1^ succinylcholine. Patients were ventilated with a bag-valve mask attached to 10 L min^−1^ oxygen. Two minutes after NMB injection, psychiatry physician placed bitemporal electrodes and applied shock as per their usual protocols for ECT (a pulse width of 1 second, pulse amplitude 800 mA, duration between 1 and 4 seconds and frequency ranges from 40 to 90 Hz; MECTA spECTrum 5000Q, Tualatin, OR, USA). 

The motor seizure duration was timed from the electric shock to the last clonic seizure activity observed in the left arm. Blood pressures, HR, and SpO_2_ were measured and recorded again after cessation of the seizure (ECT 0), 5 minutes later (ECT 5), and before leaving the recovery area (discharge). Recovery period was assessed with time to eye opening, time to following orders, and time to discharge with a Modified Aldrete Score of 8 or above.^13^ The amount of administered drugs was recorded for cost calculation.

Any adverse reactions observed during ECT procedure and recovery were recorded. Respiratory rate less than 10 per minute was recorded as respiratory depression, SpO_2_ less than 90% as hypoxemia, HR lower than 50 per minute as bradycardia, HR higher than 100 per minute as tachycardia, and MAP higher than 120 mm Hg as hypertension. Nausea, vomiting, and agitated behavior were recorded as observed.

For statistical analysis, the Statistical Package for Social Sciences version 23.0 software (IBM Corp.; Armonk, NY, USA) was used. Thirty patients for each group were calculated with an alpha error of 5% and power of 80%. Normal distribution was checked with Shapiro–Wilk test, histogram, Q-Q plot, and box plot. Mean and standard deviation were used for age, height, weight, MAP, HR, and SpO_2_. Independent sample *t*-test was used for comparison of continuous variables with normal distribution. Mann–Whitney *U* test was used for comparison of continuous variables without normal distribution. Variables within a group that changed over the course of the procedure were analyzed with Friedman repeated measures variance analysis. Nominal variables were compared with Chi-square test with Yates correction and Fisher’s exact probability test. In all previously mentioned tests, *P* < .05 was accepted as significant. Multivariable comparisons were done with the Wilcoxon test with Bonferroni’s correction (*P* < .0083 accepted as significant). 

## Results

We enrolled 60 patients scheduled for an ECT session in this study. Thirty patients were assigned to each group. The distribution of age, height, weight, and sex was similar. Group KP had 18 male and 12 female patients, group KD had 15 male and 15 female patients (*P* = .79). The average age was 45 ± 15 years in KP and 40 ± 17 years in KD (*P* = .12). Weight and height measurements were 75 ± 16 kg and 169 ± 10 cm in KP and 74 ± 15 kg and 169 ± 7 cm in KP (*P*  = .35 and .16, respectively).

**Figure 1.**
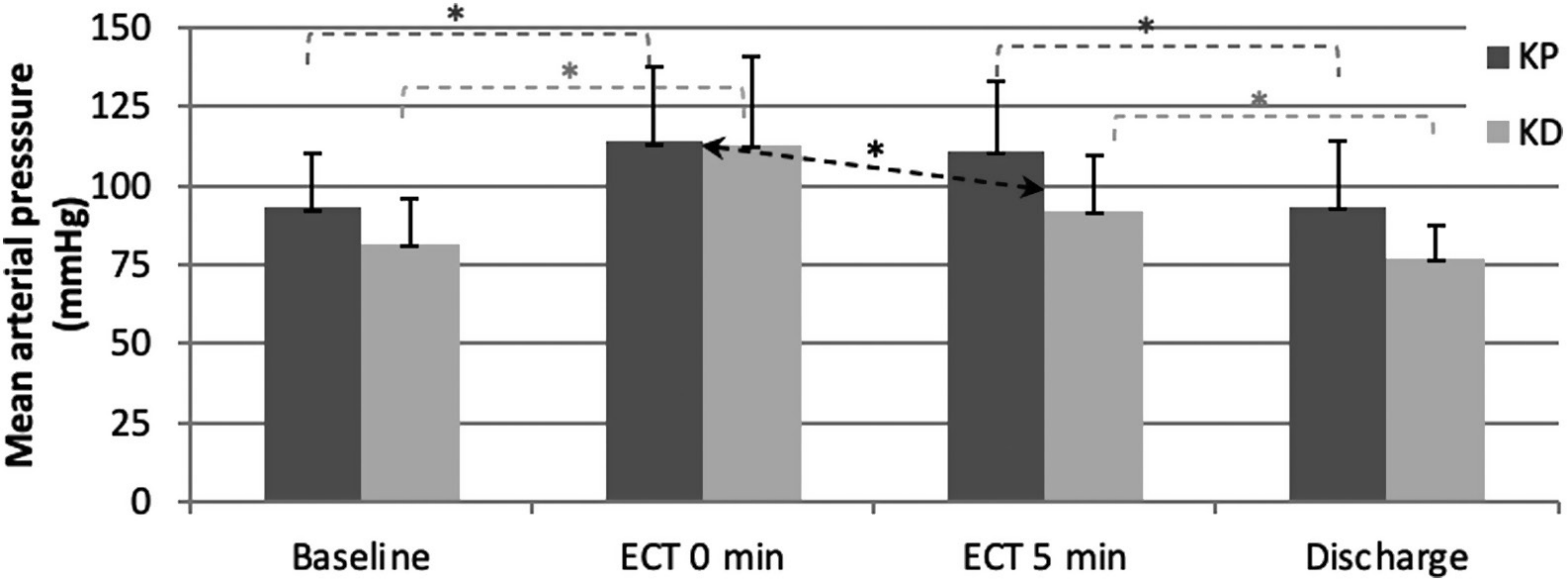
Mean arterial pressures (mean and SD, ^*^ *P *< .05). Hemodynamic response to ECT was similar in KP and KD, except from ECT 0 to ECT 5 where KD patients had a significant drop in MAP toward baseline compared to KP patients (arrow). KP, ketamine-propofol; KD, ketamine-dexmedetomidine; MAP, mean arterial pressure; SD, standard deviation, ECT, electroconvulsive therapy.

We analyzed MAP and HR to evaluate hemodynamic response and SpO_2_ for respiratory changes, and measurements in none of the parameters were normally distributed (Table 1). The change in MAP over the 4 time points were statistically significant in both groups (*P* < .001); however, the change varied slightly (Figure 1). In both groups, MAP increase from baseline to ECT 0 and MAP decrease from ECT 5 to discharge were significant (*P* < .001), while the change from baseline to discharge was insignificant (KP: *P*  = .781, KD: *P* = .094). In group KP, no significant change in MAP occurred from ECT 0 to ECT 5 (*P*  = .382), while in group KD, the drop in blood pressure was significant (*P* < .001). For the change in MAP from ECT 0 to ECT 5, the difference between 2 groups were significant (*P*  = .008). 

**Figure 2.**
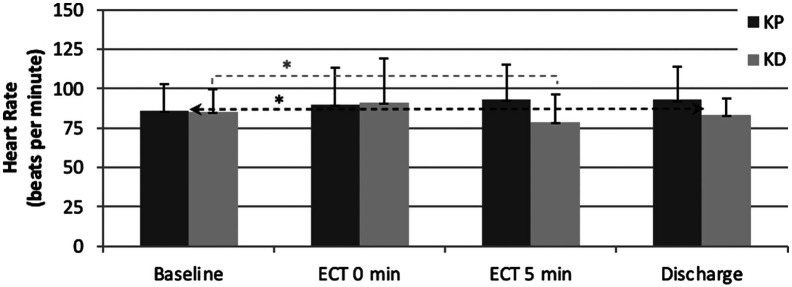
Heart rate (mean and SD, ^*^ *P *< .05). Heart rate did not change significantly over the course of ECT in both groups. The difference between the slightly increase in heart rate from baseline to discharge of KP patients and slightly decrease in heart rate (arrow). KP, ketamine-propofol; SD, standard deviation; ECT, electroconvulsive therapy.

For the group KP, the change in HR over the course of ECT was insignificant (*P*  = .3). Patients in KD had a significant change in HR over the 4 time points (*P*  = .035); however, in further analysis, only the change from baseline to ECT 5 was significant (*P*  = .006). From baseline to discharge, the HR increased in group KP and decreased in group KD, and this comparison between groups is statistically significant (*P*  = .026, Figure 2).

The change in SpO_2_ over the course of ECT was insignificant in both groups (KP: *P*  = .372, KD: *P*  = .884), and there was no significant difference between the groups.

**Figure 3.**
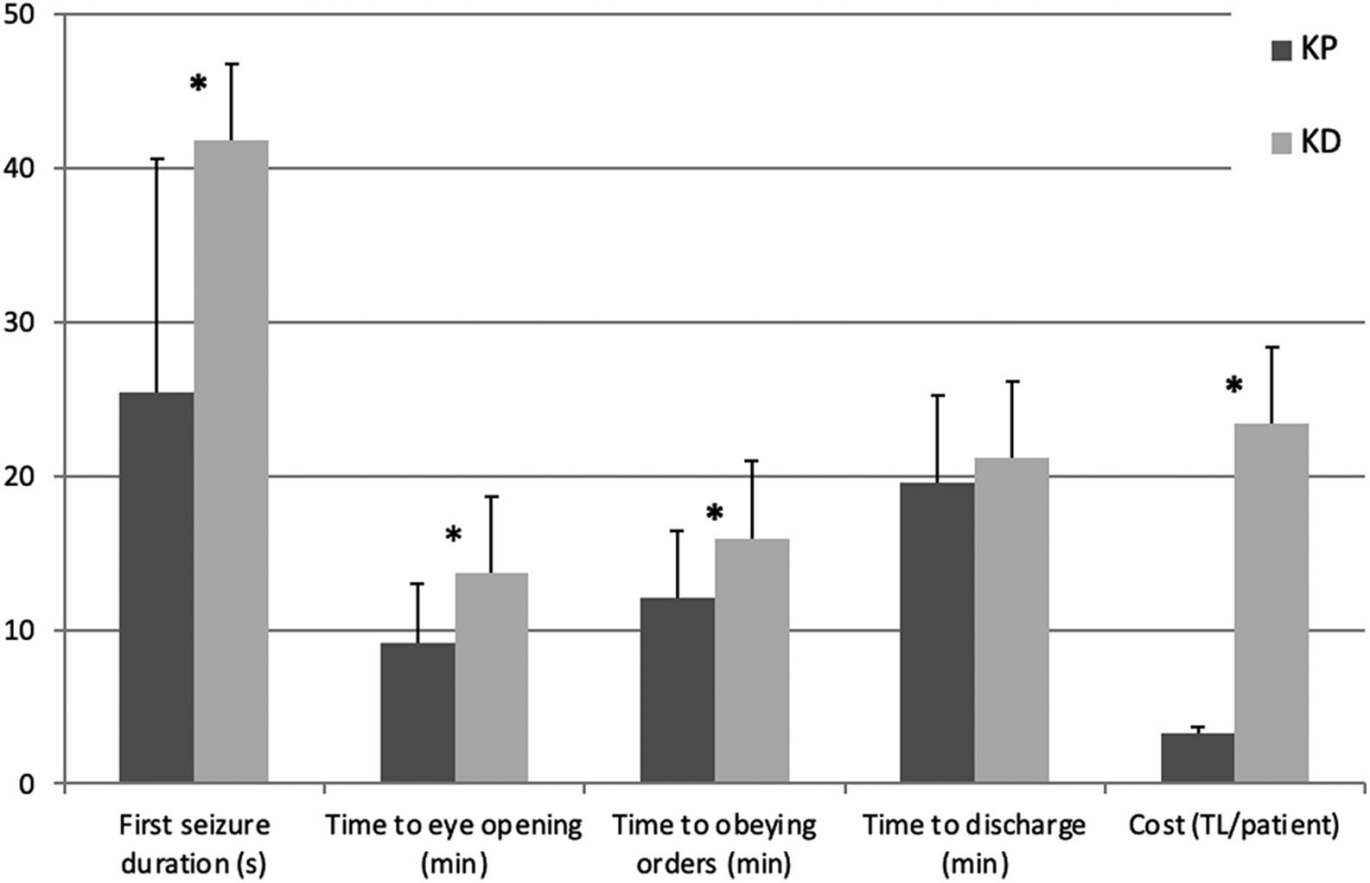
Seizure duration, recovery phase, and treatment cost (mean and SD, ^*^ *P* < .05). Seizures were longer and costs were higher for KD patients. Although KP patients started recovery earlier, all patients were ready for discharge around the same time. KP, ketamine-propofol; KD, ketamine-dexmedetomidine; SD, standard deviation.

Table 2 and Figure 3 include seizure duration, recovery phase, and cost for the treatment. The mean duration of initial seizure activity was 25.4 ± 15.2 seconds in group KP and 41.8 ± 23.0 seconds in group KD with a statistically significant difference between the 2 groups (*P * = .001). Six patients in group KP did not have adequate length seizures and the mean duration for second seizure was 26.3 ± 9.0 seconds. Two patients in group KD did not have adequate length seizures and the mean duration for second seizure was 50.5 ± 50.2 seconds. The difference in the need for a second electric shock was not statistically significant. 

Time to eye opening in group KP was significantly shorter than KD (9.1 ± 3.6 minutes vs 13.7 ± 4.4 minutes, *P* < .001). Similarly, time the patients took to obey orders was significantly shorter in group KP than group KD (12.1 ± 4.3 minutes vs 16.0 ± 4.8 minutes, *P*  = .003). Time to discharge with a modified Aldrete score of 8 or higher was similar in both groups (19.6 ± 5.7 minutes vs 21.2 ± 5.6 minutes, *P*  = .292). Treatment cost in our institution was significantly higher in group KD than KP (23.4 ± 2.4 TL vs 3.2 ± 0.5 TL per person, *P* < .001).

The difference in side effects from the 2 drug combinations was not statistically significant (*P* > .05). In group KP, 5 patients had hypertension, 3 had hypoxia, 2 had nausea, and 4 had agitation. In group KD, 5 patients had hypertension, 2 had hypoxia, 2 had nausea, 1 had vomiting, and 1 had agitation.

## Discussion

Electroconvulsive therapy is an effective therapeutic alternative in modern psychiatry for refractory depressive and psychotic disorders.^14^ General anaesthesia with muscle relaxation is essential for patient safety and well-being during ECT, and it should be carefully managed for best treatment outcomes.^2^ An ideal drug or drug combination should have no influence on seizure quality, be quick-acting, provide complete neuromuscular blockade and amnesia, and have no side effects.^5,6^ Providing satisfactory anaesthesia for an ECT patient requires a lot of tailoring from anesthesiologist’s part. In this study, we compared the KD combination to the KP combination (with succinylcholine as a neuromuscular blocker) for ECT, a comparison we did not find in the literature.

Ketamine is a commonly used agent for ECT with its anti-depressive and dissociative anaesthetic profiles and seizure lengthening effect.^15^ However, ketamine increases sympathetic discharge leading to an increase in HR and blood pressure, thus it may complicate a cardiac compromise due to ECT. Propofol is frequently used during ECT as well. Its quick onset of action and metabolism make it ideal for such a short procedure, and its anti-emetic effect can reduce postictal nausea.^16^ Its shortcomings include a decline in HR and blood pressure, respiratory depression, and a rise in seizure threshold.^7,16^ A combination of ketamine and propofol has been shown to be effective in various anaesthetic settings including ECT.^8,17^ Ketamine counteracts the anticonvulsant effect of propofol, while the 2 drugs balance out the opposite hemodynamic effects of each other.

Dexmedetomidine is becoming more popular for procedural use as it is studied further beyond intensive care.^18,19^ It provides sedation without respiratory depression, does not affect seizure duration, and blunts the sympathetic response supporting hemodynamic stability.^20^ However, adequate anaesthesia for ECT cannot be achieved solely by dexmedetomidine.^21^ Sedation is achieved quickly with KD combination, while ketamine prevents hypotension and bradycardia associated with dexmedetomidine, dexmedetomidine blunts sympathetic drive and decreases psychiatric symptoms associated with ketamine.^19^ We aimed to determine if dexmedetomidine may be superior to propofol as an adjunct to ketamine for ECT anaesthesia. 

Hemodynamic response to ECT typically consists of an initial parasympathetic response lasting 10-15 seconds followed by a sympathetic response. Cardiac complications may include left ventricular dysfunction, acute myocardial infarction, pulmonary edema, ventricular rupture, arrythmias, and asystole, especially in patients with cardiac disease.^22^ Therefore, a drug combination with the smallest hemodynamic effect is desirable. Previous studies showed that ketamine-propofol combination has better hemodynamic outcomes in terms of HR and blood pressure stability than propofol or ketamine alone during ECT.^8,10^ Similarly, a dexmedetomidine-propofol combination was shown to be superior to propofol alone in terms of hemodynamic response during ECT.^23^ Ketamine was shown to prevent dexmedetomidine-induced hypotension and bradycardia for procedural sedation.^19^

In our study, we observed an initial rise in blood pressure and reversal to baseline blood pressure level by discharge with both KP and KD patients. The change over the course of the ECT was around 20 mm Hg and statistically significant in each group. One noteworthy difference between KP and KD was that KD patients returned to near baseline blood pressure levels quicker, at 5 minutes after seizure activity. This observation is suggestive of better hemodynamic stability with ketamine and dexmedetomidine. Heart rate did not change significantly with KP and there was a slight decrease of 2 beats per minute with KD. Although statistically significant, this change is unlikely to have any clinical implications. Both combinations showed an equally good and similar heart rate response. Similarly, peripheral oxygen saturation remained unchanged with both combinations. 

Recovery from anaesthesia during the postictal phase tends to be faster with propofol alone than ketamine alone or ketamine-propofol combinations.^8,10,24^ We observed that time to eye opening and obeying orders were 4 minutes shorter in KP patients than in KD patients, showing an even longer sedation time due to dexmedetomidine. Nevertheless, time to discharge with a Modified Aldrete score of 8 or higher were similar in both groups. In practice, both combinations require similar patient observation time after ECT. 

Side effects after an ECT session include confusion, agitation, amnesia, nausea, headache, respiratory depression, and hypertension. The anaesthetic drugs contribute to these side effects as well as to induced generalized seizure. Ketamine can cause nausea and agitation and these side effects can be attenuated with propofol.^25^ Dexmedetomidine is shown to lower the incidence of postictal agitation in ECT patients.^20,26,27^ Some of our patients had nausea, vomiting, hypoxia, hypertension and/or agitation; however, we observed similar rates of side effects in each group.

Studies on anaesthetics’ effects on seizure length in ECT vary greatly in terms of the drug dosages and combinations. Although seizure duration has not been linked to treatment outcome, suppression of seizure activity by anaesthetic drugs is best avoided as a seizure shorter than 25 seconds warrants for induction of a second seizure.^3,14^ Propofol is associated with shorter seizure duration on a dose-dependent fashion; however, an unfavorable impact on clinical outcome is not proven.^9^ Nevertheless, combining propofol with ketamine can lengthen seizure length.^12^ In our study, KD patients had significantly longer seizures (41.8 seconds) than KP patients (25.4 seconds, *P*  = .001). This is a clinically relevant observation as the choice between dexmedetomidine and propofol may be made based on past seizure activity in patients with particularly short or long seizures. The number of patients who did not develop a seizure after the first electric shock was similar in both groups. This observation is in concordance with the previous observations that seizure threshold, and seizure duration has a complex relationship in the context of ECT.^28,29^

There are several limitations to our study. Although we observed longer ECT-induced seizures with KD, we did not compare the clinical outcome. We showed a slight advantage with KD in terms of hemodynamic response, yet we cannot be sure of its clinical significance without measuring clinical signs of cardiac compromise. The time points we took measurements were limited as well. An ideal study would make use of continuous blood pressure monitoring to enable recording of peak or nadir values; a non-invasive method seems to be reasonable for beat-to-beat hemodynamic monitoring during ECT.^30^ Slow injection of dexmedetomidine is a practical problem. This administration adds 10 minutes to the procedure and this delay may be unacceptable in some centers. We included the noteworthy cost difference in our results; however, we are aware that this is subject to change over time and depending on local drug prices. 

We show that dexmedetomidine is a feasible adjunct to ketamine during ECT. Ketamine-dexmedetomidine and KP combinations are comparable in terms of hemodynamic stability, recovery time, and side effect profile during ECT. Longer seizures were observed with KD compared to KP. Dexmedetomidine can complement ketamine anaesthesia for ECT with its sedative, hemodynamic and convulsive activity profile. We believe dexmedetomidine should be added to armory of anaesthetic drugs suitable for ECT, especially in patients with short seizure activity with propofol or contraindications to its use. Conversely, dexmedetomidine could be reconsidered in patients with prolonged seizures after ECT to limit seizure duration.

## Figures and Tables

**Table 1.** Mean Arterial Pressure, Heart Rate, and Peripheral Oxygen Saturation of the Patients in Groups KP and KD (Mean ± SD) and the *P*-Value for the Change Over the Course of ECT

	MAP (mm Hg)		HR (beats per minute)		SpO2 (%)	
Timing	KP	KD	KP	KD	KP	KD
Baseline	93.1 ± 16.9	81.6 ± 14.2	86.2 ± 15.8	85.4 ± 17.4	97.8 ± 1.7	99.3 ± 1.1
ECT 0	114.1 ± 23.4	112.9 ± 27.9	89.9 ± 17.2	91.3 ± 25.6	98.6 ± 7.0	99.0 ± 2.0
ECT 5	111.0 ± 21.7	91.9 ± 17.3	93.3 ± 11.4	79.0 ±14.8	98.1 ± 1.3	99.1 ± 1.7
Discharge	93.4 ± 20.9	77.0 ± 10.2	93.0 ± 15.0	83.5 ± 11.7	98.5 ± 1.1	99.0 ± 1.5
*P*	<.001	<.001	.300	.035	.372	.884

MAP, mean arterial pressure; HR, heart rate; SpO_2_, peripheral oxygen saturation; KP, ketamine-propofol; KD, ketamine-dexmedetomidine; ECT, electroconvulsive therapy.

**Table 2.** Seizure Duration, Recovery Phase, and Treatment Cost of the Patients in Groups KP and KD (Mean ± SD) and *P*-Values for Differences Between 2 Groups

	Group KP	Group KD	*P*
First seizure duration (seconds)	25.4 ± 15.2	41.8 ± 23.0	.001
Need for second shock (number)	6	2	.129
Time to eye opening (min)	9.1 ± 3.6	13.7 ± 4.4	<.001
Time to obeying orders (min)	12.1 ± 4.3	16.0 ± 4.8	.003
Time to discharge (min)	19.6 ± 5.7	21.2 ± 5.6	.292
Cost (TL/per person)	3.2 ± 0.5	23.4 ± 2.4	<.001

KP, ketamine-propofol; KD, ketamine-dexmedetomidine.

## References

[1] Weiner RD Reti IM . Key updates in the clinical application of electroconvulsive therapy. Int Rev Psychiatry. 2017;29(2):54 62. 10.1080/09540261.2017.1309362) 28406327

[2] Hermida AP Glass OM Shafi H McDonald WM . Electroconvulsive therapy in depression: current practice and future direction. Psychiatr Clin North Am. 2018;41(3):341 353. 10.1016/j.psc.2018.04.001) 30098649

[3] Rasimas JJ Stevens SR Rasmussen KG . Seizure length in electroconvulsive therapy as a function of age, sex, and treatment number. J ECT. 2007;23(1):14 16. 10.1097/01.yct.0000263254.21668.f0) 17435566

[4] Soehle M Bochem J . Anesthesia for electroconvulsive therapy. Curr Opin Anaesthesiol. 2018;31(5):501 505. 10.1097/ACO.0000000000000624) 29994943

[5] Bryson EO Aloysi AS Farber KG Kellner CH . Individualized anesthetic management for patients undergoing electroconvulsive therapy: a review of current practice. Anesth Analg. 2017;124(6):1943 1956. 10.1213/ANE.0000000000001873) 28277323

[6] Stripp TK Jorgensen MB Olsen NV . Anaesthesia for electroconvulsive therapy - New tricks for old drugs: a systematic review. Acta Neuropsychiatr. 2018;30(2):61 69. 10.1017/neu.2017.12) 28462732

[7] Rathmell JP, Rosow C. Intravenous Sedatives and Hypnotics. In: Flood P, Rathmell JP, Shafer S. Stoelting’s Pharmacology and Physiology in Anesthetic Practice. 5th ed. Wolters Kluwer; 2015.

[8] Yalcin S Aydoǧan H Selek S , et al. Ketofol in electroconvulsive therapy anesthesia: two stones for one bird. J Anesth. 2012;26(4):562 567. 10.1007/s00540-012-1378-6) 22623080

[9] Rasmussen KG Propofol for ECT anesthesia a review of the literature. J ECT. 2014;30(3):210 215. 10.1097/YCT.0000000000000093) 24820943

[10] Kayhan GE Yucel A Colak YZ , et al. Ketofol (mixture of ketamine and propofol) administration in electroconvulsive therapy. Anaesth Intensive Care. 2012;40(2):305 310. 10.1177/0310057X1204000214) 22417026

[11] Brunelin J Iceta S Plaze M , et al. The combination of propofol and ketamine does not enhance clinical responses to electroconvulsive therapy in major depression: the results from the KEOpS study. Front Pharmacol. 2020;11:562137. 10.3389/fphar.2020.562137) 33041803 PMC7522396

[12] Wang X Chen Y Zhou X Liu F Zhang T Zhang C . Effects of propofol and ketamine as combined anesthesia for electroconvulsive therapy in patients with depressive disorder. J ECT. 2012;28(2):128 132. 10.1097/YCT.0b013e31824d1d02) 22622291

[13] Aldrete JA The post-anesthesia recovery score revisited. J Clin Anesth. 1995;7(1):89 91. 10.1016/0952-8180(94)00001-K) 7772368

[14] Weiner RD Coffey CE Fochtmann LJ , et al. Practice of Electroconvulsive Therapy. Recommendations for Treatment, Training, and Privileging (A Task Force Report of the American Psychiatric Association). 2nd ed. Washington, DC: American Psychiatric Association; 2001.

[15] Jankauskas V Necyk C Chue J Chue P . A review of ketamine’s role in ECT and non-ECT settings. Neuropsychiatr Dis Treat. 2018;14:1437 1450. 10.2147/NDT.S157233) 29922060 PMC5995431

[16] Bailine SH Petrides G Doft M Lui G . Indications for the use of propofol in electroconvulsive therapy. J ECT. 2003;19(3):129 132. 10.1097/00124509-200309000-00002) 12972980

[17] Willman EV Andolfatto G . A prospective evaluation of “Ketofol” (ketamine/propofol combination) for procedural sedation and analgesia in the emergency department. Ann Emerg Med. 2007;49(1):23 30. 10.1016/j.annemergmed.2006.08.002) 17059854

[18] Gündüz M Sakalli S Güneş Y Kesiktaş E Özcengiz D Işik G . Comparison of effects of ketamine, ketamine-dexmedetomidine and ketamine-midazolam on dressing changes of burn patients. J Anaesthesiol Clin Pharmacol. 2011;27(2):220 224. 10.4103/0970-9185.81823) 21772684 PMC3127303

[19] Tobias JD Dexmedetomidine and ketamine: an effective alternative for procedural sedation? Pediatr Crit Care Med. 2012;13(4):423 427. 10.1097/PCC.0b013e318238b81c) 22067985

[20] Li X Tan F Jian CJ , et al. Effects of small-dose dexmedetomidine on hyperdynamic responses to electroconvulsive therapy. J Chin Med Assoc. 2017;80(8):476 481. 10.1016/j.jcma.2017.02.008) 28601627

[21] Shams T El-Masry R . Ketofol-Dexmedetomidine combination in ECT: a punch for depression and agitation. Indian J Anaesth. 2014;58(3):275-280. 10.4103/0019-5049.135037). PMC4090992 25024469

[22] Alpak G Ercan S Alici H , et al. Influence of recurrent electroconvulsive therapy on cardiac function. Med Princ Pract. 2014;23(3):225 228. 10.1159/000361030) 24751485 PMC5586887

[23] Begec Z Toprak HI Demirbilek S Erdil F Onal D Ersoy MO . Dexmedetomidine blunts acute hyperdynamic responses to electroconvulsive therapy without altering seizure duration. Acta Anaesthesiol Scand. 2008;52(2):302 306. 10.1111/j.1399-6576.2007.01462.x) 17976228

[24] Shah A Mosdossy G McLeod S Lehnhardt K Peddle M Rieder M . A blinded, randomized controlled trial to evaluate ketamine/propofol versus ketamine alone for procedural sedation in children. Ann Emerg Med. 2011;57(5):425 33.e2. 10.1016/j.annemergmed.2010.08.032) 20947210

[25] Strayer RJ Nelson LS . Adverse events associated with ketamine for procedural sedation in adults. Am J Emerg Med. 2008;26(9):985 1028. 10.1016/j.ajem.2007.12.005) 19091264

[26] Mizrak A Koruk S Ganidagli S Bulut M Oner U . Premedication with dexmedetomidine and midazolam attenuates agitation after electroconvulsive therapy. J Anesth. 2009;23(1):6 10. 10.1007/s00540-008-0695-2) 19234815

[27] Aksay SS Bumb JM Remennik D , et al. Dexmedetomidine for the management of postictal agitation after electroconvulsive therapy with S-ketamine anesthesia. Neuropsychiatr Dis Treat. 2017;13:1389 1394. 10.2147/NDT.S134751) 28579785 PMC5449135

[28] Chung KF Relationships between seizure duration and seizure threshold and stimulus dosage at electroconvulsive therapy: implications for electroconvulsive therapy practice. Psychiatry Clin Neurosci. 2002;56(5):521 526. 10.1046/j.1440-1819.2002.01048.x) 12193241

[29] Sackeim HA Decina P Portnoy S Neeley P Malitz S . Studies of dosage, seizure threshold, and seizure duration in ECT. Biol Psychiatry. 1987;22(3):249 268. 10.1016/0006-3223(87)90144-2) 3814678

[30] Geersing PGKB Bulte CSE Viersen VA , et al. Beat-to-beat hemodynamic monitoring during electroconvulsive therapy. J ECT. 2011;27(3):189 191. 10.1097/YCT.0b013e3182008de5) 21206372

